# 5-Aminosalicylic Acid Ameliorates Colitis and Checks Dysbiotic Escherichia coli Expansion by Activating PPAR-γ Signaling in the Intestinal Epithelium

**DOI:** 10.1128/mBio.03227-20

**Published:** 2021-01-19

**Authors:** Stephanie A. Cevallos, Jee-Yon Lee, Eric M. Velazquez, Nora J. Foegeding, Catherine D. Shelton, Connor R. Tiffany, Beau H. Parry, Annica R. Stull-Lane, Erin E. Olsan, Hannah P. Savage, Henry Nguyen, Star S. Ghanaat, Austin J. Byndloss, Ilechukwu O. Agu, Renée M. Tsolis, Mariana X. Byndloss, Andreas J. Bäumler

**Affiliations:** a Department of Medical Microbiology and Immunology, School of Medicine, University of California Davis, Davis, California, USA; b Vanderbilt Institute for Infection, Immunology, and Inflammation, Vanderbilt University Medical Center, Nashville, Tennessee, USA; c Department of Pathology, Microbiology, and Immunology, Vanderbilt University Medical Center, Nashville, Tennessee, USA; Weill Cornell Medical College

**Keywords:** dysbiosis, *Escherichia coli*, gut inflammation, inflammatory bowel disease, microbial communities

## Abstract

5-Aminosalicylic acid (5-ASA), a peroxisome proliferator-activated receptor gamma (PPAR-γ) agonist, is a widely used first-line medication for the treatment of ulcerative colitis, but its anti-inflammatory mechanism is not fully resolved. Here, we show that 5-ASA ameliorates colitis in dextran sulfate sodium (DSS)-treated mice by activating PPAR-γ signaling in the intestinal epithelium. DSS-induced colitis was associated with a loss of epithelial hypoxia and a respiration-dependent luminal expansion of Escherichia coli, which could be ameliorated by treatment with 5-ASA. However, 5-ASA was no longer able to reduce inflammation, restore epithelial hypoxia, or blunt an expansion of E. coli in DSS-treated mice that lacked *Pparg* expression specifically in the intestinal epithelium. These data suggest that the anti-inflammatory activity of 5-ASA requires activation of epithelial PPAR-γ signaling, thus pointing to the intestinal epithelium as a potential target for therapeutic intervention in ulcerative colitis.

## Abstract (precis)

An expansion of *Enterobacterales* in the fecal microbiota is a microbial signature of dysbiosis that is linked to many noncommunicable diseases, including ulcerative colitis. Here, we used Escherichia coli, a representative of the *Enterobacterales*, to show that its dysbiotic expansion during colitis can be remediated by modulating host epithelial metabolism.

## INTRODUCTION

Ulcerative colitis is thought to result from an inappropriate microbiota-driven activation of the mucosal immune system (1). The fecal microbiota in healthy subjects is dominated by obligate anaerobic bacteria belonging to the classes *Clostridia* (phylum *Firmicutes*) and *Bacteroidia* (phylum *Bacteroidetes*) (2). Ulcerative colitis is associated with an elevated relative abundance of facultative anaerobic bacteria belonging to the *Enterobacterales* (ord. nov. [3], phylum *Proteobacteria*) (4–6), a change in the microbiota composition that can exacerbate colitis in mouse models (7, 8). However, it remains unclear which host cells initiate a microbiota-driven activation of the mucosal immune system during ulcerative colitis.

Clinical trials in the 1960s established that sulfasalazine, which is composed of sulfapyridine linked by an azo bond to 5-aminosalicylic acid (5-ASA), can bring mild to moderate cases of ulcerative colitis into remission (9, 10). The therapeutically active moiety of sulfasalazine is 5-ASA (11), an agonist of peroxisome proliferator-activated receptor gamma (PPAR-γ) (12). PPAR-γ is a member of the nuclear receptor superfamily (13), which is expressed in several cell types implicated in the pathogenesis of ulcerative colitis, including macrophages, T cells, and the colonic epithelium (14). Treatment with 5-ASA ameliorates 2,4,6-trinitrobenzene sulfonic acid (TNBS)-induced colitis in wild-type mice, but not in whole-body heterozygous *Pparg^+/−^* mice (12). However, the cell type in which PPAR-γ arbitrates its anti-inflammatory activity during ulcerative colitis remains a point of contention.

5-ASA is thought to act locally in the colon (15); epithelial cells from ulcerative colitis patients exhibit lower PPAR-γ synthesis than healthy subjects (16, 17). In rodent models of dextran sulfate sodium (DSS)-induced colitis, 5-ASA restores PPAR-γ synthesis in the colonic mucosa (18) and ameliorates the severity of inflammation (19). Mice lacking *Pparg* expression in both epithelial cells and hemopoietic cells (*Pparg^fl/fl^ Mmtv^cre+^* mice) are more sensitive to TNBS-induced colitis (20). Based on *in vitro* evidence showing that activation of PPAR-γ in regulatory T cells (T_regs_) downregulates CD4 effector T cell functions, enhanced colitis in *Pparg^fl/fl^ Mmtv^cre+^* mice is proposed to be due to lack of *Pparg* expression in T_regs_ (20). Another PPAR-γ agonist, rosiglitazone, can also ameliorate DSS-induced colitis in mice (21). However, while mice lacking *Pparg* expression specifically in the intestinal epithelium still respond to rosiglitazone treatment (21), the drug can no longer ameliorate DSS-induced colitis in mice lacking *Pparg* expression specifically in macrophages (22). Collectively, these data seem to raise questions about the importance of colonic epithelial cells in producing the anti-inflammatory activity of 5-ASA.

In addition to ameliorating inflammation, treatment with 5-ASA reduces the abundance of *Enterobacterales*, such as Escherichia coli, in the fecal microbiota of ulcerative colitis patients (23). Based on the observation that 5-ASA can inhibit the growth of E. coli in the test tube, it has been postulated that 5-ASA might directly block the growth of *Enterobacterales* to abrogate their proinflammatory activity (24). Alternatively, 5-ASA might check the growth of *Enterobacterales* during colitis by activating PPAR-γ in macrophages, T cells, or the colonic epithelium. These alternate hypotheses can be explored using mice with DSS-induced colitis, because this model responds to 5-ASA treatment (18, 19) and features a marked expansion of E. coli in the colonic microbiota (25). The goal of this study was to determine the role of the colonic epithelium in a 5-ASA-mediated block of an E. coli expansion in the gut microbiota during DSS-induced colitis.

## RESULTS

### 5-ASA checks the growth of E. coli during DSS-induced colitis.

We first determined whether the ability of 5-ASA to reduce the abundance of E. coli in the fecal microbiota of ulcerative colitis patients (23) could be recapitulated during DSS-induced colitis in mice. To establish an animal model for addressing this question, we used C57BL/6J mice from The Jackson Laboratory, which do not carry endogenous *Enterobacterales* (26), because the vendor screens against the presence of this taxon in its special-pathogen-free procedures. The fact that the ecological niche of *Enterobacterales* remains unoccupied during microbiota assembly in C57BL/6J mice offers the unique opportunity for precision editing of the microbiota by the designed engraftment of E. coli strains engineered to probe the contribution of predestined metabolic pathways to bacterial growth. Since previous studies suggested that an E. coli expansion in DSS-treated mice required genes for the respiration of host-derived oxygen and nitrate (25, 27, 28), C57BL/6J mice that were confirmed to be *Enterobacterales* free were engrafted with a 1:1 mixture of a respiration-proficient E. coli strain (Nissle 1917, wild type) and an isogenic mutant lacking genes for aerobic and nitrate respiration (*cydA napA narG narZ* mutant). Differential recovery of these strains serves as an indicator for the bioavailability of respiratory electron acceptors in the colon (29). DSS treatment of male and female mice was associated with reduced colon length (see Fig. S1A in the supplemental material) and a marked E. coli expansion (Fig. S1B), which required genes for aerobic and nitrate respiration, as indicated by increased recovery of the wild type over that of the *cydA napA narG narZ* mutant from DSS-treated mice in a comparison with controls (Fig. S1C).

10.1128/mBio.03227-20.1 FIG S1 DSS treatment induces respiration-dependent E. coli expansion in male and female mice. Groups of male and female C57BL/6J mice (*N *= 6) receiving drinking water supplemented with DSS (DSS) or no drinking water supplementation (Mock) were engrafted with E. coli strains (a 1:1 mixture of the wild type and *cydA napA narG narZ* mutant). (A) Colon length was determined at necropsy. (B) Numbers of CFU of E. coli Nissle 1917 recovered from colon contents. (C) Competitive indices (ratio of the E. coli Nissle 1917 wild type to an isogenic *cydA napA narG narZ* mutant) in colon contents were determined. *, *P < *0.05; **, *P < *0.01; ***, *P < *0.001. Download FIG S1, PDF file, 0.02 MB. Copyright © 2021 Cevallos et al. 2021 Cevallos et al. This content is distributed under the terms of the Creative Commons Attribution 4.0 International license.

**FIG 1:**
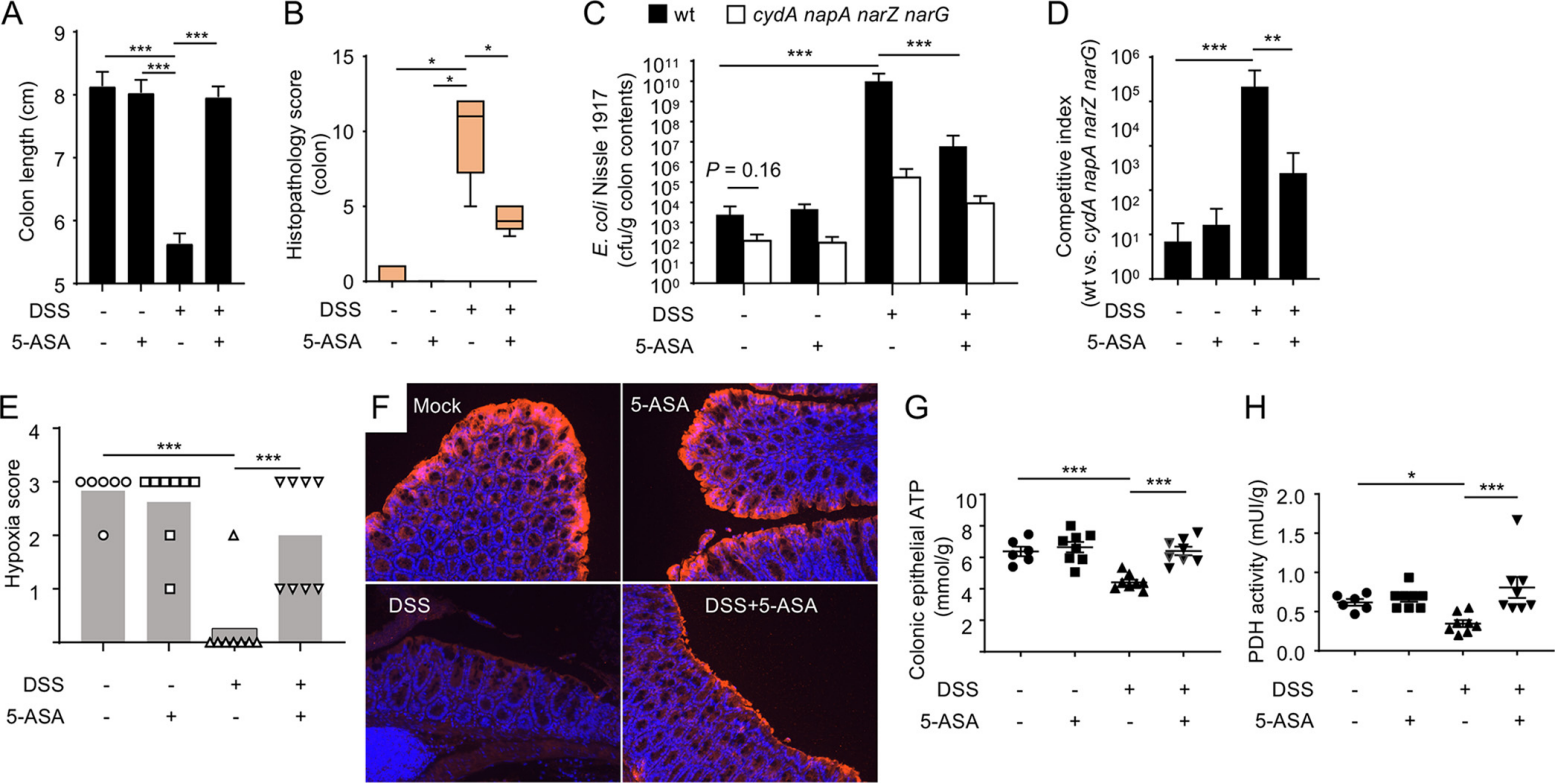
5-ASA blocks E. coli expansion during DSS-induced colitis. Groups of male C57BL/6J mice (*N* is indicated in panel E) receiving conventional chow (5-ASA, –) or chow supplemented with 5-ASA (5-ASA, +) and drinking water supplemented with DSS (DSS, +) or no drinking water supplementation (DSS, –) were engrafted with E. coli strains (a 1:1 mixture of the wild type [wt] and *cydA napA narG narZ* mutant). (A) Colon length was determined during necropsy. (B) A veterinary pathologist scored histopathological changes in blinded sections of the colon. (C) Numbers of CFU of E. coli Nissle 1917 recovered from colon contents. (D) Competitive index (ratio of the E. coli Nissle 1917 wild type to an isogenic *cydA napA narG narZ* mutant) in colon contents was determined. (E and F) Binding of pimonidazole was detected using Hypoxyprobe-1 primary antibody and a Cy3-conjugated goat anti-mouse secondary antibody (red fluorescence) in the sections of the proximal colon that were counterstained with DAPI nuclear stain (blue fluorescence). (E) Pimonidazole staining was quantified by scoring blinded sections of the proximal colon. (F) Representative images are shown. (G and H) Colonocytes were isolated from the colonic mucosa to measure cytosolic concentrations of ATP (G) or pyruvate dehydrogenase (PDH) activity (H). *, *P < *0.05; **, *P < *0.01; ***, *P < *0.001.

Having established a model to study an expansion of E. coli during DSS-induced colitis, we wanted to determine whether the growth of this species could be limited by 5-ASA treatment. DSS treatment resulted in marked colitis, as indicated by a reduction in colon length (Fig. 1A) and marked histopathological lesions (Fig. 1B), including crypt hyperplasia, an epithelial repair response, and lower numbers of alcian-blue positive (goblet) cells (Fig. S2A and S2B), which is reflective of a reduction in the number of terminally differentiated cells during epithelial repair. However, these signs of disease were blunted in animals receiving 5-ASA supplementation (Fig. 1A and B; Fig. S2A and S2B). DSS treatment resulted in a marked expansion of E. coli in the colonic microbiota, which was blunted by 5-ASA treatment (Fig. 1C). As in a previous report (24), 5-ASA reduced the growth of E. coli under aerobic conditions *in vitro* (Fig. S2C). However, 5-ASA did not inhibit the growth of E. coli under anaerobic conditions (Fig. S2D), suggesting that environmental factors markedly influenced the inhibitory activity of 5-ASA, thus making *in vitro* growth assays a poor predictor of whether the drug would inhibit bacterial growth in the intestine *in vivo.* Notably, 5-ASA supplementation in mock-treated mice did not reduce the overall recovery of E. coli from colon contents compared to that from mock-treated mice without 5-ASA supplementation (Fig. 1C), which did not support the idea that 5-ASA directly acts on bacteria to block the growth of E. coli in the colon (24). In conclusion, 5-ASA treatment checked an E. coli expansion in mice with DSS-induced colitis, thus recapitulating a reduction in the E. coli abundance observed in ulcerative colitis patients during 5-ASA therapy (23).

10.1128/mBio.03227-20.2 FIG S2 5-ASA ameliorates signs of disease and aerobic E. coli expansion during DSS-induced colitis. Groups of male C57BL/6J mice receiving conventional chow (5-ASA, –) or chow supplemented with 5-ASA (5-ASA, +) and drinking water supplemented with DSS (DSS, +) or no drinking water supplementation (DSS, –) were engrafted with a 1:1 mixture of the E. coli wild type and *cydA napA narG narZ* mutant (A and B) or a 1:1 mixture of the E. coli wild type and a *cydA* mutant (E and F). (A and B) Goblet cells were visualized histologically by alcian blue staining, which is specific for sulfated and carboxylated acid mucopolysaccharides and sulfated and carboxylated sialomucins. Representative images (A) and a quantification of the number of goblet cells (B) are shown. (C and D) The growth of E. coli Nissle 1917 in medium without supplementation (mock) or in medium with the indicated concentration of 5-ASA (between 0.25 and 2 mg 5-ASA/ml) was followed over time under aerobic (C) or anaerobic (D) growth conditions by measuring the optical density of the culture at 600 nm. (E) Colon length was determined at necropsy. (F) Competitive indices (ratio of the E. coli Nissle 1917 wild type to an isogenic *cydA* mutant) in colon contents were determined. *, *P < *0.05; **, *P < *0.01; ***, *P < *0.001. Download FIG S2, PDF file, 1.6 MB. Copyright © 2021 Cevallos et al. 2021 Cevallos et al. This content is distributed under the terms of the Creative Commons Attribution 4.0 International license.

Elevated recovery of the respiration-proficient E. coli wild type over the respiration-deficient *cydA napA narG narZ* mutant was consistent with previous reports indicating that DSS-induced colitis increases the luminal bioavailability of oxygen and nitrate (25, 27, 28), but this increase in the competitive index was blunted in DSS-treated mice receiving 5-ASA supplementation (Fig. 1D). These data suggested that 5-ASA supplementation checks an E. coli expansion by limiting the availability of host-derived electron acceptors. The colonic epithelium is a potential source of host-derived oxygen, because epithelial oxygenation increases during DSS-induced colitis (28). Visualization of epithelial oxygenation with pimonidazole, a 2-nitroimidazole that is reductively activated specifically in hypoxic cells (<1% oxygen) (30, 31), revealed that DSS treatment eliminated epithelial hypoxia in the colon, whereas 5-ASA supplementation restored epithelial hypoxia in DSS-treated mice (Fig. 1E and F). Since PPAR-γ maintains epithelial hypoxia in the colon by increasing oxygen consumption in the mitochondria (29), we investigated whether 5-ASA supplementation restored markers of mitochondrial bioenergetics in the colonic epithelium. Consistent with impaired mitochondrial bioenergetics, DSS treatment reduced epithelial ATP levels (Fig. 1G) and decreased epithelial levels of pyruvate dehydrogenase activity (Fig. 1H). Levels of these markers of mitochondrial bioenergetics could be restored in the colonic epithelium by 5-ASA treatment (Fig. 1G and H). To directly address the contribution of oxygen, mice were engrafted with a 1:1 mixture of a respiration-proficient E. coli strain (Nissle 1917, wild type) and an isogenic mutant lacking *cydA*, the gene encoding cytochrome *bd* oxidase, which is required for aerobic respiration under microaerophilic conditions (29). DSS-induced colitis (Fig. S2E) provided a *cydA*-dependent fitness advantage, which was blunted by treatment with 5-ASA (Fig. S2F).

**FIG 2:**
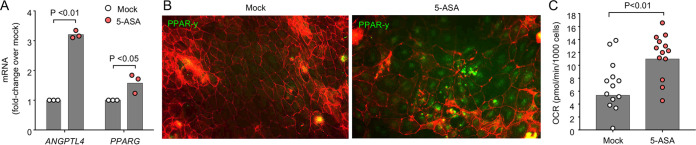
5-ASA stimulates oxygen consumption in CaCo-2 cells. CaCo-2 cells were mock treated or treated with 5-ASA. (A) The transcript levels of the indicated genes were determined by quantitative real-time PCR. (B) The synthesis of PPAR-γ was detected by fluorescence microcopy using an anti-PPAR-γ antibody (green fluorescence) and staining of actin, with phalloidin as a counterstain (red fluorescence). (C) The oxygen consumption rate (OCR) of CaCo-2 cells was determined using an Agilent Seahorse XFe96 analyzer. (A and C) Each dot represents data from one well.

In conclusion, the ability of 5-ASA to check aerobic-respiration-dependent (Fig. S2F) E. coli expansion (Fig. 1C) correlated with a return of homeostatic functions of epithelial cells in DSS-treated mice, including restoration of epithelial hypoxia (Fig. 1E and F), and reestablished mitochondrial bioenergetics in the colonic epithelium (Fig. 1G and H). We thus wanted to determine whether 5-ASA directly activates PPAR-γ in colonic epithelial cells to check the growth of E. coli. Stimulation of cultured human colonic cancer epithelial (CaCo-2) cells with 5-ASA induced expression of *PPARG* and the PPAR-γ-activated gene *ANGPTL4* (Fig. 2A) and triggered synthesis and the nuclear localization of PPAR-γ (Fig. 2B). Although there was no significant effect on either basal or maximal mitochondrial respiration (data not shown), 5-ASA stimulation increased the spare mitochondrial respiratory capacity in CaCo-2 cells (Fig. 2C), which was indicative of increased mitochondrial bioenergetics in response to cellular stress (i.e., inflammation).

### 5-ASA activates epithelial PPAR-γ signaling to check the growth of E. coli during colitis.

**FIG 3:**
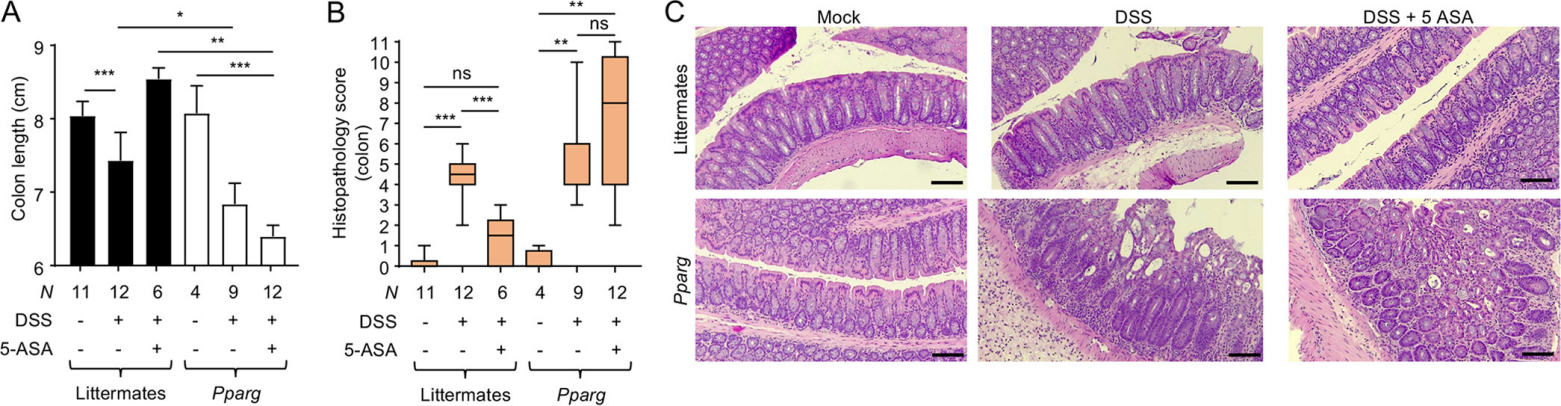
5-ASA ameliorates DSS-induced colitis by activating epithelial PPAR-γ signaling. Groups of *Pparg^fl/fl^ Villin^cre^*^/−^ mice that lack PPAR-γ signaling in the intestinal epithelium (*Pparg*) and littermate *Pparg^fl/fl^ Villin*^−/−^ control mice (Littermates) received conventional chow (5-ASA, –) or chow supplemented with 5-ASA (5-ASA, +) and drinking water supplemented with DSS (DSS, +) or no drinking water supplementation (DSS, –). (A) Colon length was determined during necropsy. (B and C) Blinded sections of the colon from each animal were evaluated by a veterinary pathologist. (B) Histopathology score. The boxes in the whisker blot represent the first to third quartiles, and the mean value of the gross pathology scores is indicated by a line. (C) Representative images of colonic sections for each group are shown. The scale bars represent 300 μm. *, *P < *0.05; **, *P < *0.01; ***, *P < *0.001; ns, differences were not significant.

To investigate the importance of epithelial PPAR-γ signaling as a target for 5-ASA therapy *in vivo*, we generated mice lacking PPAR-γ specifically in the intestinal epithelium (*Pparg^fl/fl^ Villin^cre^*^/–^ mice) along with wild-type littermate control animals (*Pparg^fl/fl^ Villin^−/−^* mice) using mice obtained from The Jackson Laboratory that were confirmed to be *Enterobacterales* free. Mice were engrafted with indicator strains for assessing the bioavailability of respiratory electron acceptors in the colon (i.e., a 1:1 mixture of E. coli strain Nissle 1917 and an isogenic *cydA napA narG narZ* mutant). Consistent with a previous report (21), mice lacking epithelial PPAR-γ signaling were more susceptible to DSS-induced colitis, as indicated by a worsened shortening of the colon (Fig. 3A) and elevated histopathology scoring (Fig. 3B and C). Notably, 5-ASA treatment ameliorated signs of inflammation in DSS-treated littermate control mice, but not in DSS-treated mice lacking epithelial PPAR-γ signaling (Fig. 3A to D), pointing to the colonic epithelium as a key player in negotiating the anti-inflammatory activity of 5-ASA.

**FIG 4:**
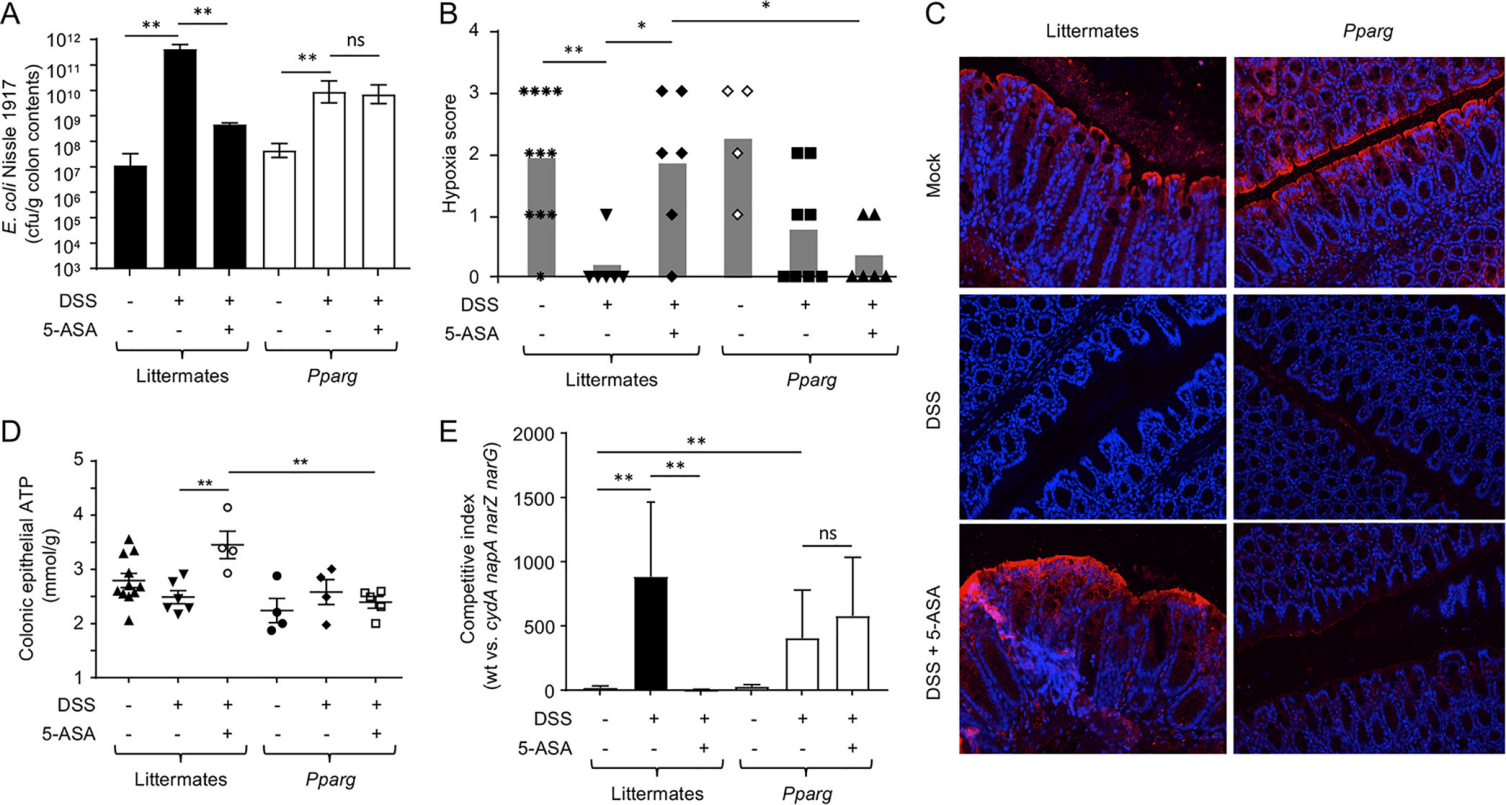
5-ASA restores epithelial hypoxia and ameliorates dysbiotic E. coli expansion by activating epithelial PPAR-γ signaling. Groups (*N* is indicated in panel B) of *Pparg^fl/fl^ Villin^cre^*^/−^ mice that lack PPAR-γ signaling in the intestinal epithelium (*Pparg*) and littermate *Pparg^fl/fl^ Villin*^−/−^ control mice (Littermates) receiving conventional chow (5-ASA, –) or chow supplemented with 5-ASA (5-ASA, +) and drinking water supplemented with DSS (DSS, +) or no drinking water supplementation (DSS, –) were engrafted with E. coli strains (a 1:1 mixture of the wild type and *cydA napA narG narZ* mutant). (A) Numbers of CFU of E. coli Nissle 1917 recovered from colon contents. (B and C) Binding of pimonidazole was detected using Hypoxyprobe-1 primary antibody and a Cy3-conjugated goat anti-mouse secondary antibody (red fluorescence) in the sections of proximal colon that were counterstained with DAPI nuclear stain (blue fluorescence). (B) Pimonidazole staining was quantified by scoring blinded sections of the proximal colon. (C) Representative images are shown. (D) Colonocytes were isolated from the colonic mucosa to measure cytosolic concentrations of ATP. (E) Competitive indexes (ratio of the E. coli Nissle 1917 wild type to an isogenic *cydA napA narG narZ* mutant) in colon contents were determined. *, *P < *0.05; **, *P < *0.01; ***, *P < *0.001; ns, *P > *0.05.

Importantly, 5-ASA treatment blunted DSS-induced E. coli expansion in the colon only in littermate control mice, not in mice lacking epithelial PPAR-γ signaling (Fig. 4A). These data did not support the idea that 5-ASA is a direct inhibitor of E. coli growth *in vivo* (24) but instead suggested that the drug checks the growth of *Enterobacterales* indirectly by activating PPAR-γ signaling in the host epithelium. Further analysis of the consequences of epithelial PPAR-γ signaling revealed that 5-ASA treatment restored epithelial hypoxia in DSS-treated littermate control mice but not in DSS-treated mice lacking epithelial PPAR-γ signaling (Fig. 4B and C), suggesting that 5-ASA acts on the epithelium to restore mitochondrial bioenergetics. Consistently with this idea, colonic epithelial ATP levels and PDH levels were reinstated by 5-ASA supplementation in DSS-treated littermate control mice but not in DSS-treated mice lacking epithelial PPAR-γ signaling (Fig. 4D and Fig. S3A). Finally, 5-ASA supplementation diminished the respiration-dependent growth advantage of wild-type E. coli over the growth of a *cydA napA narG narZ* mutant in DSS-treated littermate control mice but not in DSS-treated mice lacking epithelial *Pparg* expression (Fig. 4E), which was consistent with the proposed role of epithelial PPAR-γ signaling in limiting the availability of respiratory electron acceptors in the colonic lumen to check the growth of facultative anaerobic *Enterobacterales* (29).

10.1128/mBio.03227-20.3 FIG S3 5-ASA normalizes epithelial pyruvate dehydrogenase activity by activating epithelial PPAR-γ signaling. Groups (*N* is indicated by the number of dots) of *Pparg^fl/fl^ Villin^cre^*^/−^ mice that lack PPAR-γ signaling in the intestinal epithelium (*Pparg*) and littermate *Pparg^fl/fl^ Villin*^−/−^ control mice (Littermates) receiving conventional chow (5-ASA, –) or chow supplemented with 5-ASA (5-ASA, +) and drinking water supplemented with DSS (DSS, +) or no drinking water supplementation (DSS, –) were engrafted with E. coli strains (a 1:1 mixture of the wild type and a *cydA napA narG narZ* mutant). Colonocytes were isolated from the colonic mucosa to measure cytosolic concentrations of pyruvate dehydrogenase (PDH) activity. **, *P < *0.01. Download FIG S3, PDF file, 0.02 MB. Copyright © 2021 Cevallos et al. 2021 Cevallos et al. This content is distributed under the terms of the Creative Commons Attribution 4.0 International license.

## DISCUSSION

Previous work in the DSS colitis model suggests that the PPAR-γ agonist rosiglitazone ameliorates colitis by activating PPAR-γ signaling in macrophages (22), whereas epithelial *Pparg* expression is not required for its anti-inflammatory activity (21). In contrast, the PPAR-γ agonist 5-ASA ameliorated DSS-induced colitis by activating PPAR-γ signaling in the intestinal epithelium. In healthy volunteers, a sizable fraction of orally administered 5-ASA reaches the distal colon, as indicated by fecal secretion of approximately 15% of the dose, which can be further increased to 37 to 58% of fecal secretion after administration of azo compounds, such as sulfasalazine, or slow-release compounds, such as mesalazine, which is ethylcellulose-coated 5-ASA (32). Conversely, orally administered rosiglitazone is completely absorbed (99%), and a lack of fecal secretion suggests that the drug does not reach the colonic lumen (33). Thus, differences in pharmacokinetics might help explain why the anti-inflammatory activity of rosiglitazone involves activation of PPAR-γ signaling in macrophages (22), whereas 5-ASA activated PPAR-γ signaling in the colonic epithelium to ameliorate DSS-induced colitis. Rosiglitazone is effective in treating mild to moderate cases of ulcerative colitis (34), but in combination with 5-ASA, rosiglitazone achieves better therapeutic effects (35). The observation that rosiglitazone and 5-ASA activate PPAR-γ signaling in different cell types to ameliorate DSS-induced colitis might explain why, paradoxically, a combination of two PPAR-γ agonists has therapeutic effects that are superior to those of treatment with just one agonist.

Azo compounds (e.g., olsalazine and balsalazide) or slow-release compounds (e.g., mesalazine) of 5-ASA are first-line therapeutics for the induction of remission and maintenance in patients with mild to moderate ulcerative colitis (36), but the anti-inflammatory mechanism of 5-ASA remains unclear. Putative anti-inflammatory activities of 5-ASA include decreasing NF-κB activation in the nucleus (37) and inhibiting prostaglandin production in the intestinal mucosa (38) by activating PPAR-γ signaling (12). In addition to having an anti-inflammatory activity, PPAR-γ has an important role in regulating cellular energy metabolism by activating mitochondrial bioenergetics (39). PPAR-γ-mediated activation of mitochondrial bioenergetics in colonic epithelial cells increases oxygen consumption, thereby rendering the epithelial surface hypoxic (40, 41). In turn, epithelial hypoxia limits the amount of oxygen emanating from the mucosal surface, thereby checking the growth of facultative anaerobic *Enterobacterales*, which can use this resource to outgrow obligate anaerobic bacteria in the gut microbiota (29). Here, we show that 5-ASA restored epithelial hypoxia in DSS-treated mice and limited an aerobic-respiration-dependent expansion of E. coli in the colonic microbiota by activating epithelial PPAR-γ signaling. These data help explain why treatment with 5-ASA reduces the abundance of E. coli in the fecal microbiotas of ulcerative colitis patients (23).

Whereas 5-ASA can induce remission in patients with mild to moderate ulcerative colitis, the drug is no longer therapeutically active during severe acute ulcerative colitis, and corticosteroids become the mainstay of therapy (42). A possible reason why 5-ASA is no longer useful in patients with severe acute ulcerative colitis is that other cell types, such as T cells, might become more important in driving inflammation during later stages of disease, thus rendering a treatment that targets solely the epithelium ineffective. Conversely, the finding that 5-ASA activates epithelial PPAR-γ signaling to ameliorate DSS-induced colitis in mice points to the colonic epithelium as an important driver of inflammation during mild to moderate ulcerative colitis.

## MATERIALS AND METHODS

### Contact for reagent and resource sharing.

Further information and requests for resources and reagents should be directed to the lead contact, Andreas J. Bäumler.

### Experimental model and subject details. (i) Bacterial strains and culture conditions.

E. coli Nissle 1917 is a commensal human isolate (43) that has been marketed as a probiotic. The E. coli Nissle 1917 *cydA napA narG narZ* mutant and the E. coli Nissle 1917 *cydA* mutant used in this study have been described previously (29). E. coli strains were routinely grown aerobically at 37°C in LB broth (BD Biosciences) or on LB agar plates. When appropriate, antibiotics were added to the medium at the following concentrations: 0.1 mg/ml carbenicillin and 0.05 mg/ml kanamycin. For competitive infection experiments, strains were grown in a hypoxia chamber (0.8% oxygen) at 37°C in LB broth (BD Biosciences).

### (ii) Caco-2 cell culture.

Caco-2 cells (ATCC; HTB-37) were cultured in minimal essential medium (MEM) (Gibco; 11090099) supplemented with 10% fetal bovine serum (FBS) (Gibco; 16140071), 1% GlutaMAX-I (Gibco; 35050061), 1% MEM nonessential amino acids (Gibco; 11140-030), and 1 mM sodium pyruvate (Gibco; 11360-070).

### (iii) Animal experiments.

All experiments in this study were approved by the Institutional Animal Care and Use Committee at the University of California Davis. Female and male C57BL/6J mice, aged 8 to 10 weeks, were obtained from The Jackson Laboratory. Female and male C57BL/6 *Pparg^fl/fl^ Villin^cre–^* and littermate *Pparg^fl/fl^ Villin^−/−^* (control) mice were generated at UC Davis by mating *Pparg^fl/fl^* mice with *Villin^cre/–^* mice (The Jackson Laboratory).

*(a) DSS treatment.* Male and female C57BL/6J mice were given 3% DSS (Alfa Aesar) in their drinking water continuously for 8 days. At day 4 of DSS treatment, mice were inoculated with 1 × 10^9^ CFU of a 1:1 mixture of the above-indicated E. coli strains. Samples were collected at 4 days postinfection.

*(b) 5-ASA treatment.* Male C57BL/6J mice were given chow (Envigo, Taklad) supplemented with 2.5 g 5-ASA (Sigma-Aldrich)/kg of body weight for 9 to 10 days. At day 3 of 5-ASA supplementation, mice were given 2.5% DSS in their drinking water for the remainder of the experiment. At day 4 of DSS treatment, mice were inoculated with 1 × 10^9^ CFU of a 1:1 mixture of the above-indicated E. coli strains. Samples were collected either 2 or 3 days after infection.

*(c) Experiments with Pparg^fl/fl^ Villin^cre^*^/−^ *mice.* Female and male *Pparg^fl/fl^ Villin^cre^*^/−^ mice and littermate *Pparg^fl/fl^ Villin*^−/−^ control mice, aged 8 to 14 weeks, were given chow supplemented with 2.5 g/kg 5-ASA for 9 days. At day 3 of supplementation, mice were given 2.5% in their drinking water for the remainder of the experiment. At day 4 of DSS treatment, mice were inoculated with 1 × 10^9^ CFU of a 1:1 mixture of the above-indicated E. coli strains. Samples were collected at 2 days after infection.

### Method details. (i) Colonocyte isolation.

The colon and part of the cecum were opened lengthwise and cut into 2- to 4-cm pieces, collected in 15 ml of ice-cold 1× RPMI 1640 buffer (Gibco) in a 50-ml Falcon tube, and cleaned with 20 ml of ice-cold 1× Dulbecco’s phosphate-buffered saline (DPBS; Gibco) in another 50-ml Falcon tube. The tissue was then placed into 15-ml conical centrifuge tubes filled with 10 ml of ice-cold dissociation reagent 1 (30 mM EDTA, 1.5 mM dithiothreitol [DTT], diluted into 1× DPBS) and placed on ice for 20 min. Tissues were then placed into a 15-ml conical centrifuge tube filled with 6 ml of warm (37°C) dissociation reagent 2 (30 mM EDTA, diluted into 1× DPBS) and incubated for 10 min at 37°C. After this incubation, tubes were shaken vigorously for 30 s to detach the epithelium from the basement membrane, for a total of about 80 to 90 shake cycles. Remnant intestinal tissue was removed, and the pellet cell solution was centrifuged at 800 × *g* for 5 min at 4°C. The supernatant was removed, and the cell pellet was resuspended in 1 ml of Tri reagent (Molecular Research Center) for subsequent RNA extraction or in radioimmunoprecipitation assay (RIPA) buffer for metabolism analysis.

### (ii) ATP measurements.

For measuring intracellular ATP levels, primary colonocytes were isolated as described above and then deproteinized by using a deproteinizing sample preparation kit (BioVision, Milpitas, CA) according to the manufacturer’s instructions. Lactate measurements in colonocyte lysates were performed by using an ATP colorimetry assay kit (BioVision, Milpitas, CA) according to the manufacturer’s instructions.

### (iii) PDH activity measurements.

To measure PDH activity, primary colonocytes were isolated as described above and then lysed with RIPA buffer, and cellular debris were removed by centrifugation for 5 min at 13,000 rpm at room temperature. PDH activity was measured with supernatants by using the PDH activity assay kit (BioVision, Milpitas, CA) according to the manufacturer’s instructions.

### (iv) Histopathological analysis.

Colonic tissues were fixed in 10% phosphate-buffered formalin, and 5-μm sections of the tissue samples were stained with hematoxylin and eosin (H&E). Representative images were taken using an Olympus BX41 microscope at a magnification of ×10. Scoring of blinded tissue sections was performed by a veterinary pathologist based on the criteria listed in Table S2 in the supplemental material.

### (v) Alcian blue staining.

Colon and cecal tissues were fixed in 10% phosphate-buffered formalin, and 5-μm sections of the tissue samples were stained with alcian blue. Pictures were captured on an AxioCam camera (2.2-μm by 2.2-μm pixel distance). Quantification of mature mucus-producing, alcian blue-positive cells was conducted based on a comet-like feature of the cells, with dense blue staining on the apical side of colonic crypt within the frame. Each measurement was conducted based on 5 frames per individual mouse; the average for each group was based on data from six mice per group determined at a magnification of ×40.

### (vi) Hypoxia staining.

For detection of hypoxia, mice were treated with 60 mg/kg of pimonidazole HCl intraperitoneally (Hypoxyprobe-1 kit; Hypoxyprobe) 1 h prior to euthanasia. Colon samples were fixed in 10% phosphate-buffered formalin, and paraffin-embedded tissue was blocked with mouse-on-mouse blocking reagent (Vector Labs) and probed with mouse anti-pimonidazole monoclonal IgG1 (monoclonal antibody 4.3.11.3). Then, slides were stained with Cy3-conjugated goat anti-mouse antibody (Jackson ImmunoResearch Laboratories). Samples were counterstained with 4′,6′-diamidino-2-phenylindole (DAPI) using SlowFade Gold mountant. Samples were scored based on the degree of colonic epithelial hypoxia (0, no hypoxia; 1, mild focal hypoxia; 2, moderate multifocal hypoxia; 3, intense diffuse hypoxia). Representative images were obtained using a Zeiss Axiovert 200 M fluorescence microscope and were brightness adjusted.

### (vii) Fluorescence microscopy.

Caco-2 cells were cultured in T25 flasks as described previously. After reaching confluence, Caco-2 cells were harvested from each flask and resuspended in 15 ml MEM. Autoclaved glass coverslips were placed into a 24-well plate, and 500 μl of Caco-2 cell suspension was added to each well. After 48 h of incubation, Caco-2 cells were treated with 15 mM 5-ASA (Acros Organics) for 24 h. Cells were then washed 1× in PBS and permeabilized with 0.2% Triton X-100 for 2 min on ice. Permeabilized cells were treated with 4% paraformaldehyde for 20 min at room temperature and then washed with PBS. Ammonium chloride (NH_4_Cl) was then added to cells for 15 min. Afterwards, cells were treated with 0.05% Triton X-100 for 10 min on ice. The surface was blocked by 10% goat serum with 0.3 M glycine for 1 h at room temperature. Subsequently, the primary anti-PPAR-γ antibody (1:100; Santa Cruz Biotechnology) was incubated overnight at 4°C. The secondary antibody (1:1,000; BD Pharmingen) was then incubated for 1 h at room temperature. Finally, the cells were incubated with phalloidin for labeling actin filaments. Between each step, the cells were washed in PBS. The stained cells were imaged using a Zeiss Axioplan 2 microscope with DAPI-FITC (fluorescein isothiocyanate)-TRITC (tetramethyl rhodamine isocyanate) excitation filter sets. Images were analyzed using ImageJ software.

### (viii) Caco-2 RNA extraction and quantitative reverse transcription-PCR.

For gene expression assays, Caco-2 cells were grown until they were 70% confluent and then seeded into 6-well plates. After 48 h, 15 mM 5-ASA (Acros Organics) was added to cells and incubated for 6 h. Cells were then washed with DPBS, 0.05% trypsin-EDTA (Gibco) was added, and cells were harvested. Cell pellets were stored at −80°C. RNA was isolated according to the protocol provided (Norgen Biotek Corporation) and stored at −80°C. RNA was reverse transcribed using an iScript gDNA Clear cDNA synthesis kit (Bio-Rad). Quantitative PCR was performed using SYBR green (SsoAdvanced; Bio-Rad) for PPAR-γ and ANGPTL4. Primers are listed in Table S1. The expression of target genes was normalized to that of 18S rRNA.

10.1128/mBio.03227-20.4 TABLE S1 Primers used in this study. Download Table S1, PDF file, 0.02 MB. Copyright © 2021 Cevallos et al. 2021 Cevallos et al. This content is distributed under the terms of the Creative Commons Attribution 4.0 International license.

10.1128/mBio.03227-20.5 TABLE S2 Criteria for histopathology scoring. Download Table S2, PDF file, 0.01 MB. Copyright © 2021 Cevallos et al. 2021 Cevallos et al. This content is distributed under the terms of the Creative Commons Attribution 4.0 International license.

### (ix) Caco-2 cell measurement of oxygen consumption rates.

Experiments were performed using the Seahorse XF Cell Mito stress test kit (Agilent; 103708-100) according to the manufacturer’s instructions and carried out using an Agilent Seahorse XFe96 analyzer. Two days prior to 5-ASA treatment, Caco-2 cells were seeded in an XF cell culture microplate (Agilent) and placed in a tissue culture incubator at 37°C. One day prior to the assay, a utility plate (Agilent) was hydrated overnight with tissue culture-grade water at 37°C in a non-CO_2_ incubator. Immediately prior to the running of the assay, water was removed from the utility plate, replaced with 200 μl of prewarmed 37°C XF calibrant (Agilent), and placed in a non-CO_2_ incubator for 45 min to 1 h. Also immediately prior to the running of the assay, spent culture medium was replaced with 180 μl Seahorse XF Dulbecco’s modified Eagle’s medium (DMEM), pH 7.4 (Agilent; 103575-100) supplemented with 5.5 mM glucose (Agilent; 103577-100), 1% GlutaMAX-I, and 1 mM sodium pyruvate (Agilent complete medium), and cells were placed in a non-CO_2_ incubator at 37°C for 1 h. Stock compounds were prepared and loaded into the sensor cartridge (Agilent) ports according to the manufacturer’s instructions, with maintenance of a final well concentration of 1.0 μM oligomycin, 0.25 μM FCCP {2-[2-[4-(trifluoromethoxy)phenyl]hydrazinylidene]-propanedinitrile}, and 0.5 μM rotenone/antimycin A.

### (x) Bacterial growth assay.

To determine how 5-ASA affects the growth of E. coli Nissle 1917 *in vitro*, LB broth was prepared with increasing amounts of 5-ASA. 5-ASA (Sigma-Aldrich) was dissolved directly in LB broth at a final concentration of 2 mg/ml. Sodium hydroxide (Honeywell) was added to the medium until 5-ASA dissolved. LB broth containing 2 mg/ml 5-ASA was diluted in LB broth to final 5-ASA concentrations of 1 mg/ml and 0.25 mg/ml. The pH of LB broth alone and LB broth plus 5-ASA were matched to pH 7.0 using hydrochloric acid or sodium hydroxide and measured using pH strips. Overnight cultures of E. coli Nissle 1917 were harvested, washed in PBS, and added to a final optical density at 600 nm (OD_600_) of 0.0001 in LB broth alone or LB broth containing 5-ASA. For anaerobic growth assays, the OD_600_ was measured every hour for 24 h using Epoch 2 plate reader (BioTek) at 37°C with shaking. For aerobic growth assays, cultures were grown at 37°C with shaking and the OD_600_ was measured at 4, 8, 12, and 24 h.

### (xi) Quantification and statistical analysis.

To analyze ratios (i.e., competitive indices and fold changes in mRNA levels), bacterial numbers were transformed logarithmically prior to statistical analysis. An unpaired Student *t* test was used on the transformed data to determine whether differences between groups were statistically significant (*P < *0.05). To determine the statistical significance of differences in total histopathology scores, a Mann-Whitney *U* test was used.
