# Supplementary figures and images for: 5-Aminosalicylic Acid Ameliorates Colitis and Checks Dysbiotic Escherichia coli Expansion by Activating PPAR-γ Signaling in the Intestinal Epithelium
Source: mBio. 2021 Jan 19;12(1):e03227-20. doi: 10.1128/mBio.03227-20 (PMC7845635; doi:10.1128/mBio.03227-20)

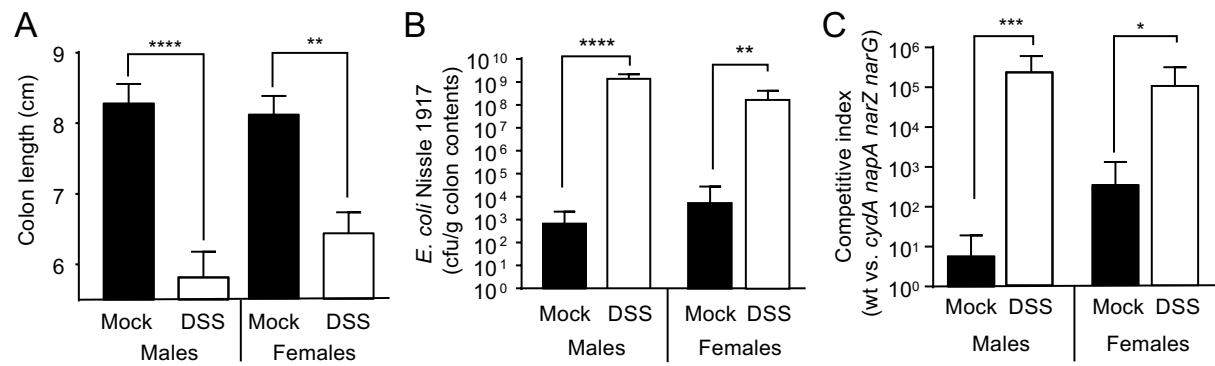

Fig S1

Supplement: FIG S1 [file mBio.03227-20-sf001.pdf]

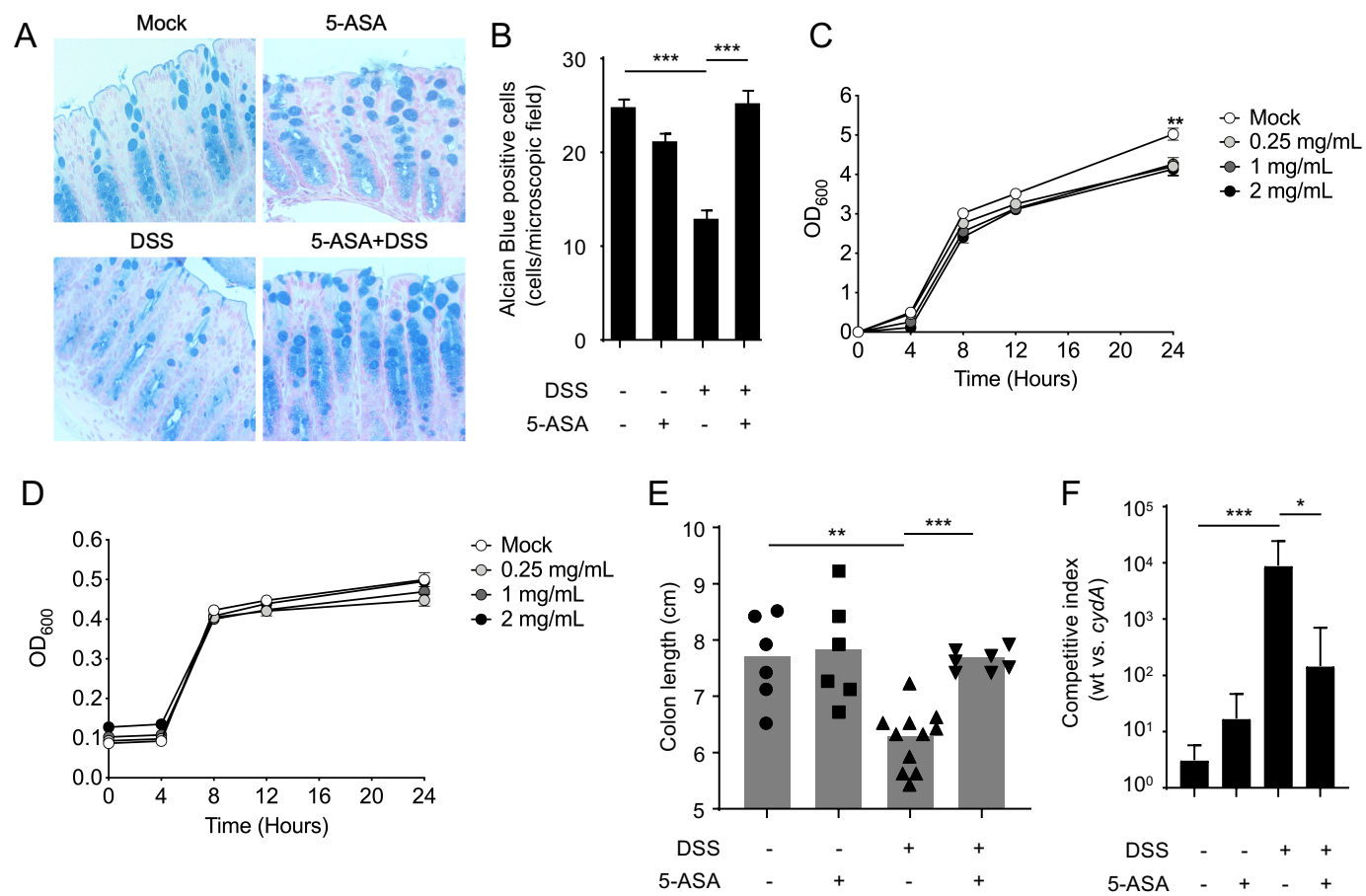

Fig S2

Supplement: FIG S2 [file mBio.03227-20-sf002.pdf]

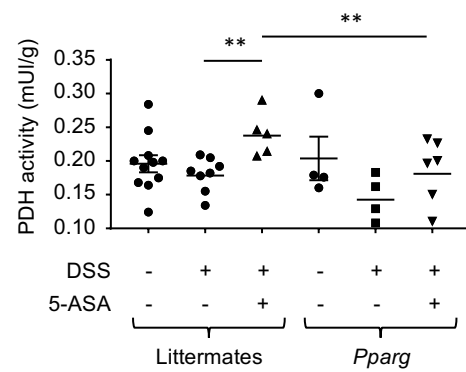

Figure S3

Supplement: FIG S3 [file mBio.03227-20-sf003.pdf]
