# Supplementary material for: 5-Aminosalicylic Acid Ameliorates Colitis and Checks Dysbiotic Escherichia coli Expansion by Activating PPAR-γ Signaling in the Intestinal Epithelium
Source: mBio. 2021 Jan 19;12(1):e03227-20. doi: 10.1128/mBio.03227-20 (PMC7845635; doi:10.1128/mBio.03227-20)
Supplement: TABLE S1 [file mBio.03227-20-st001.pdf]

Supplementary Table 1: Primers used in this study

Genes	Forward	Reverse
PPARG (human)	5'- TACTGTCGGTTTCAGAAATGCC -3'	5'- GTCAGCGGACTCTGGATTCAG -3'
ANGPTL4 (human)	5'- CACAGCCTGCAGACACAACTC -3'	5'- GGAGGCCAAACTGGCTTTGC -3'
18S(human)	5'- GATATGCTCATGTGGTGTTGA -3'	5'- ACGTTCCACCTCATCCTCA -3'
