# Supplementary material for: 5-Aminosalicylic Acid Ameliorates Colitis and Checks Dysbiotic Escherichia coli Expansion by Activating PPAR-γ Signaling in the Intestinal Epithelium
Source: mBio. 2021 Jan 19;12(1):e03227-20. doi: 10.1128/mBio.03227-20 (PMC7845635; doi:10.1128/mBio.03227-20)
Supplement: TABLE S2 [file mBio.03227-20-st002.pdf]

Supplementary Table 2: Criteria for histopathology scoring

Score	Infiltration inflammatory cells	Submucosal edema	Epithelial damage	Exudate
3	Severe multifocal to diffuse infiltration	Severe (>40%)	Multifocal to diffuse ulceration and/or severe multifocal to diffuse enterocyte hyperplasia with metaplasia	Severe accumulation
2	Moderate multifocal to diffuse infiltration	Moderate (20-40%)	Moderate multifocal to diffuse loss of goblet cells associated with enterocyte hyperplasia	Moderate accumulation
1	Mild multifocal diffuse infiltration	Mild (1-20%)	Erosion/mild loss of goblet cells and enterocyte hyperplasia	Mild accumulation
0	No infiltration	Absent	Absent	Absent
